# Metal-on-Metal Hip Arthroplasty: A Review of Adverse Reactions and Patient Management

**DOI:** 10.3390/jfb6030486

**Published:** 2015-06-26

**Authors:** James Drummond, Phong Tran, Camdon Fary

**Affiliations:** 1 Melbourne Medical School, University of Melbourne, Melbourne, Victoria 3052, Australia; 2 Department of Orthopaedics, Western Health, Melbourne, Victoria 3011, Australia; E-Mails: office@phongtran.com.au (P.T.); camfary@gmail.com (C.F.)

**Keywords:** metal-on-metal, hip arthroplasty, hip replacement, hip resurfacing, adverse reactions, patient management, pseudotumour, aseptic lymphocyte-dominated vasculitis-associated lesion (ALVAL), revision surgery

## Abstract

Recent alarming joint registry data highlighting increased revision rates has prompted further research into the area of metal-on-metal hip replacements and resurfacings. This review article examines the latest literature on the topic of adverse reactions to metal debris and summarises the most up-to-date guidelines on patient management. Adverse reactions to metal debris can cause significant damage to soft tissue and bone if not diagnosed early. Furthermore, not every patient with an adverse reaction to metal debris will be symptomatic. As such, clinicians must remain vigilant when assessing and investigating these patients in order to detect failing implants and initiate appropriate management.

## 1. Background

Total hip arthroplasty (THA), performed most commonly for advanced osteoarthritis, is a highly successful procedure believed to have originated in Germany in 1891 [1]. Since then, a number of different bearing components have been utilised in an effort to improve survivorship and reduce complications. While metal-on-polyethylene (MoP) bearings are currently the most widely implanted prosthesis, recently, metal-on-metal (MoM) bearings have come under the spotlight amid evidence of higher than expected revision rates.

Since 2008, it has become evident from national joint registry data that hip replacements and resurfacings with MoM bearing surfaces have significantly higher revision rates compared to those with MoP [2]. Approximately 1 in 5 MoM hip replacements will need revision 10–13 years after they were implanted, with larger head sizes (≥ 36 mm) ascribing higher risk [2,3]. This is compared with MoP implants, which are revised in less than 4% of cases 10 years after insertion [3]. Furthermore, approximately 13% of hip resurfacing operations will require revision 10 years after the primary surgery [3]. With the release of this information and the subsequent recalls of the DePuy Articular Surface Replacement (ASR) and Zimmer Durom hip resurfacing systems, the number of MoM hip replacements and resurfacings has reduced dramatically since 2007. MoM hip replacements now account for less than 1% of all hip arthroplasty procedures in Australia and the UK [3,4]. 

The increased revision rates of MoM hip replacements are thought to relate to adverse reactions to metal debris released from the bearing surface as the implant wears. These reactions cause soft tissue changes in the surrounding tissues, known as “pseudotumours”. While these lesions can be locally destructive to nearby muscle and bone, many patients are asymptomatic, making identification of these reactions difficult in a number of cases. As such, several government authorities have published nationally and internationally recognised guidelines to assist patients, general practitioners and surgeons on the use and monitoring of MoM bearings for total hip arthroplasty and hip resurfacing.

This review will cover the assessment and management of patients who have undergone a metal-on-metal total hip arthroplasty or resurfacing procedure. We will summarise the currently accepted guidelines for managing those who are not only presenting with potential joint failure, but also those who are asymptomatic. A clear summary of these guidelines can be found in Table 1. This table is based on the final opinion statement on metal-on-metal (MoM) hip arthroplasty published by the Scientific Committee on Emerging and Newly Identified Health Risks in September 2014 [5], the Therapeutic Goods Administration information statement on MoM hip replacements updated in March 2015 [6] and the Medicines and Healthcare Products Regulatory Agency medical device alert on MoM hip replacements published in June 2012 [7]. Clinicians are reminded to carefully consider every patient with a MoM implant on an individual basis as symptoms and pathology may differ. 

**Table 1:** Guidelines for patient management.

Clinical Consideration	Small Head MoM THA (femoral head < 36mm)		Large Head MoM THA (femoral head ≥ 36mm)		Hip Resurfacing Arthroplasty	
	Asymptomatic	Symptomatic	Asymptomatic	Symptomatic	Asymptomatic	Symptomatic
Imaging Required	MARS MRI or USS if concern or clinical/blood metal ion abnormality)	Plain X-ray and MARS MRI or USS	MARS MRI or USS if concern clinical/blood metal ion abnormality)	Plain X-ray and MARS MRI or USS	MARS MRI or USS if concern or clinical/blood metal ion abnormality)	Plain X-ray and MARS MRI or USS
Blood metal ion testing (cobalt and chromium)	Performed at each follow-up	Performed at and between each follow-up	Performed at each follow-up	Performed at and between each follow-up	Performed at each follow-up	Performed at and between each follow-up
Abnormal blood metal ions	Repeat test after 3 months if concentration >7 μg/L (or >2 μg/L with concerns)	Repeat test after 3 months if concentration >7 μg/L (or >2 μg/L with concerns)	Repeat test after 3 months if concentration >7 μg/L (or >2 μg/L with concerns)	Repeat test after 3 months if concentration >7 μg/L (or >2 μg/L with concerns)	Repeat test after 3 months if concentration >7 μg/L (or >2 μg/L with concerns)	Repeat test after 3 months if concentration >7 μg/L (or >2 μg/L with concerns)
Follow up timeframe	As per local protocol for conventional THA	No less than annually for life of MoM implant	Annually for life of MoM implant	No less than annually for life of MoM implant	Annually for first 5 years, then as per local protocol for conventional THA (annual for life of implant if ≤50mm diameter, female or low coverage arc)	No less than annually for life of MoM implant
When to consider revision	Cross-sectional imaging abnormalities and/or where blood Co/Cr levels progressively rising > 7 μg/L	Persistent symptoms, cross-sectional imaging abnormalities and/or where blood Co/Cr levels progressively rising > 7 μg/L	Cross-sectional imaging abnormalities and/or where blood Co/Cr levels progressively rising > 7 μg/L	Persistent symptoms, cross-sectional imaging abnormalities and/or where blood Co/Cr levels progressively rising > 7 μg/L	Cross-sectional imaging abnormalities and/or where blood Co/Cr levels progressively rising > 7 μg/L	Persistent symptoms, cross-sectional imaging abnormalities and/or where blood Co/Cr levels progressively rising

## 2. What Is a Metal-on-Metal Hip Replacement?

Metal-on-metal relates to the composition of the articulating bearing surface used in two specific joint replacement surgeries currently being performed.

In conventional metal-on-metal total hip replacement surgery, a large metal femoral head on a standard femoral stem articulates with a metal acetabular cup or liner. This differs slightly to hip resurfacing, where a similar metal acetabular cup articulates with a large metal femoral head or cap that is cemented on to the native bone of the femoral neck.

## 3. What Is the Rationale for Metal-on-Metal Articulating Surfaces?

Metal articulating surfaces were initially utilised as they could be engineered to be extremely smooth and hard, two properties that reduce wear rate. The wear rate of MoM implants is estimated to be 60 times less than conventional MoP implants [8]. A lower wear rate is expected to prolong the life of the implant, delaying the need for any revision surgery. As such, MoM hip replacements and resurfacings have traditionally been used in younger, more active patients.

Other reported advantages of MoM hip arthroplasty include an increased resistance to dislocation associated with large diameter femoral heads and reduced osteolysis due to the absence of polyethylene particles [8]. Furthermore, hip resurfacing has the benefit of bone conservation, which is believed to lead to improved revision surgery outcomes, especially for femoral components [9,10].

Unfortunately, the trade-off for utilising metal components is the potential for an increased release of metal ions, namely cobalt (Co) and chromium (Cr), which can adversely react with local tissues.

## 4. What Are the Issues Specific to Metal-on-Metal Articulating Surfaces?

Soft tissue inflammatory reactions to metal debris are a recognised complication of metal-on-metal hip arthroplasty. These reactions, which are grouped under the umbrella term adverse reactions to metal debris (ARMD), have been called inflammatory pseudotumour, aseptic lymphocytic vasculitis associated lesion (ALVAL) and metallosis. The spectrum of ARMD is extensive and ranges from small asymptomatic cysts to large soft tissue masses (pseudotumours) [5].

Inflammatory pseudotumour is the clinical term given to an aseptic mass in the periprosthetic tissues that is either solid or cystic and is associated with clinical, radiological or histopathological signs of inflammation. It is thought to develop as a result of an adverse reaction to the metal ions released from the wear of metal articular bearing surfaces. The specific design characteristics of the metal prostheses are believed to contribute to different wear patterns and hence different amounts of metal debris released into the periprosthetic tissues. It is known that implanting the acetabular cup at abnormal inclination angles creates greater edge loading, leading to increased prosthetic wear [11]. Furthermore, shallow acetabular cups, such as those utilised in the Depuy ASR hip resurfacing device, exhibit greater edge loading, leading to increased wear [12].

ALVAL is a histological diagnosis that describes the unique cellular changes that occur periprosthetically in response to metal particles, namely cobalt and chromium ions. The mechanism is believed to be a T lymphocyte mediated type IV hypersensitivity reaction, with tissue damage occurring as a result of cytotoxic T cells and activated monocytes/macrophages [13]. The presence of this reaction is thought to be proportional to the amount of wear debris released, but has also been observed in patients with smaller amounts of wear debris [12].

Metallosis, defined as aseptic fibrosis, local necrosis or loosening of a device secondary to metallic corrosion and release of wear debris, is commonly found in tissue samples from joints exhibiting an ARMD [12]. It has been suggested that pseudotumours are on the same pathological spectrum of disease as metallosis, and will develop if given enough time [12].

A recently proposed theory is that trunnionosis, defined as metal wear at the head-neck junction of a total hip implant, is an important contributor (along with wear at the bearing surfaces) to metal particle release in metal-on-metal total hip arthroplasty [14,15]. Trunnionosis is a recognised issue with implants employing a modular design, which are often used to improve prosthetic flexibility when attempting to restore normal hip biomechanics. Trunnionosis occurs similarly in modular MoM and MoP total hip replacements as both head-neck junctions consist of metal on metal surfaces. If wear patterns at the head-neck junction are similar between implants, however, it can be assumed that trunnionosis cannot be the sole cause of increased rates of adverse reactions in MoM hip replacements. The importance of this phenomenon was recognised recently after two modular implant designs were recalled due to higher than expected fretting and corrosion and hence, increased risk of ARMD [16]. As modular junction designs are not used in hip resurfacing, this phenomenon is only observed in total hip replacements.

## 5. How Common Are Adverse Reactions?

Due to the variability in presentations of adverse reactions to metal debris, they are often difficult to diagnose. Asymptomatic pseudotumours have been identified on ultrasound and MRI scanning in 27%–32% of patients with MoM hip replacements [17,18] There is conflicting evidence, however, on whether or not these asymptomatic pseudotumours grow in size to a point where revision is necessary [18,19]

A case study of 1224 patients in 2009 concluded that female patients and those under the age of 40 had significantly increased risk of revision due to pseudotumour [20]. Australian National Joint Registry data highlight the significance of this, with the number of hip resurfacing operations being performed on female patients dramatically reducing since 2007, to the point where only 1% of hip resurfacing operations were performed on women in 2013 [4]. A recent report on 706 hips, however, found no association between age and sex and pseudotumour formation [21]. 

While the incidence of these adverse reactions may be greater than that predicted by the revision rate, data reported by national joint registries currently provide us with the best idea of their frequency. The latest data from the National Joint Registry for England, Wales and Northern Ireland reveal that the 10 year revision rate for cemented and uncemented MoP total hip replacements is 3.13% and 3.98% respectively [3]. This is compared to MoM bearing replacements, which have 19.68% and 21.92% revision rates for cemented and uncemented procedures respectively. The Australian National Joint Registry data also reflect this, documenting a 13-year cumulative revision rate of 19.4% for all MoM total hip replacements, as opposed to 6.8% for all bearing surfaces excluding large head (≥ 36mm) MoM [4]. Metal related pathology has been reported to account for nearly 40% of all MoM surface revisions [2].

The size of the prosthesis is an important consideration, with 25.5% of all MoM total hip replacements utilising a head >40mm in size being revised within 10 years [2]. This is compared to those with a head size ≤28mm, of which 5.7% require revision within 10 years.

With regards to resurfacing, different brands of prosthesis have different revision probabilities at 10 years. The 10-year revision probability of the ASR system is reported at 30.36%, compared to the Birmingham Hip Resurfacing system (BHR), at 9.04% [3].

## 6. Are There Any Other Concerns?

Due to the renal excretion of Co-Cr ions, there has been concern regarding potential for increased risk of bladder and other cancers in patients with metal-on-metal hip replacements. A large observational study performed on nearly 300,000 patients with total hip replacements in England and Wales found that patients with MoM hip replacements were not at higher risk of developing cancer compared to those with alternative bearings [22]. Another cohort study in Finland examining nearly 29,000 patients who had undergone total hip replacement concluded that the overall risk of cancer or death is not increased after MoM hip replacement compared with conventional total hip replacement [23]. The follow-up periods for these studies were seven and four years respectively. While long term follow-up studies are limited, a recent case series of 49 patients reviewed 17 years after MoM hip arthroplasty demonstrated a cancer incidence in the MoM hip replacement group similar to that of the general population [24]. Additional research documenting long-term outcomes of large cohorts must be performed before confidently declaring that MoM hip replacements are not associated with an increased risk of cancer.

Because metal-on-metal hip replacements and resurfacings are often used in women of child-bearing age, it is important to consider the potential effects of metal ions during maternity. It has been shown that while metal ion degradation products from MoM implants do cross the placenta, 15% of maternal chromium and 50% of maternal cobalt are filtered, resulting in reduced amounts being passed to the foetus [25]. A number of case series have documented no negative effects on foetal or child development because of abnormally high maternal cobalt or chromium ion levels secondary to MoM arthroplasty [26,27,28]. 

## 7. How Do Patients with Adverse Reactions to Metal Debris Present Clinically?

Patients with adverse reactions to metal debris commonly present with pain, usually located in the groin, with occasional radiation to the greater trochanter and down the thigh [12]. Pain is thought to be the strongest predictor for pseudotumour presence [21]. This pain will often cause patients to adopt an antalgic gait. Over time, this may progress to instability with or without dislocation and patients may complain of clicking or clunking sensations in the hip. Patients may also experience other symptoms such as stiffness, reduced range of movement, abductor weakness and even rash as a reaction to the metal ions.

Other complications associated with hip resurfacings and large diameter MoM hip replacements may present with similar symptoms. As such, it is vital to attain a complete history, perform a thorough clinical examination and conduct relevant investigations in order to rule out other potential diagnoses.

Any patient presenting with a symptomatic hip resurfacing or metal on metal hip replacement requires immediate referral to an orthopaedic specialist for thorough examination and investigation. Because of the destructive nature of some pseudotumours, it is vital that this occurs without delay. Furthermore, due to the high rate of asymptomatic pseudotumours, it is important that general practitioners remain vigilant when examining any patient who has previously undergone a MoM bearing hip replacement or resurfacing.

## 8. Excluding Other Diagnoses

While metal related pathology is the most common reason for revision of MoM primary hip replacements, it is also important to rule out other causes of hip arthroplasty failure such as component loosening, infection, periprosthetic fracture and acute dislocation [2]. Referred pain from the spine or pelvis should also be considered.

Anteroposterior radiographs of the pelvis and hip are a cheap and effective way of ruling out periprosthetic fracture (which may occur in the femoral neck after resurfacing procedures) and acute dislocation. Comparing sequential radiographic images with those taken immediately after implantation will also allow for detection of loosening or migration of components.

Due to the potentially devastating consequences of infection in patients who have undergone hip arthroplasty, it is imperative to exclude infection in patients presenting with local signs such as erythema, warmth or swelling or systemic signs and symptoms. While C reactive protein (CRP) and erythrocyte sedimentation rate (ESR) are traditionally used and have very high specificity in MoP hip arthroplasty, there have been cases of elevated ESR and CRP in patients with MoM hip replacements exhibiting non-infected adverse soft tissue reactions [29]. As such, interpretation of these results in patients with MoM total hip replacements must be done with caution. Furthermore, synovial fluid white cell counts can also be raised in patients presenting with adverse soft tissue reactions to MoM hip replacements. Despite these reports, we still recommend full blood examination with a white cell count and differentiation as well as CRP/ESR for baseline blood investigations.

## 9. Should We Be Testing Blood Metal Ion Concentrations?

A recent systematic review examining results from 9957 patients concluded that metal ion concentrations, particularly cobalt and chromium, are consistently higher than baseline following metal-on-metal hip replacement or resurfacing [30]. In addition, patients with MoP or MoC hip replacements had significantly lower blood ion concentrations than those with MoM bearings.

Threshold limits for metal ion concentrations in well-functioning *versus* poorly functioning implants have been investigated by a number of researchers. Günther *et al*. demonstrated from 8 case series that, despite a large spread of cobalt ion concentrations, mean values for well-functioning implants seemed to lie within a limited range (1.5–3.5 μg/L) [31]. Even though mean concentrations for poorly functioning implants varied more widely (2.1–29.7 μg/L), on average, these levels were considerably higher compared with those functioning well. Furthermore, patients with pseudotumours after implantation of an ASR hip resurfacing device had higher cobalt and chromium ion concentrations than those without pseudotumours (cobalt median 8.3 μg/L *versus* 1.0 μg/L, chromium median 5.9 μg/L *versus* 1.3 μg/L) [32]. These papers indicate that while setting a “cut-off” metal ion concentration level for poorly functioning implants is difficult, there may be a correlation between higher metal ion concentrations and implants which are functioning poorly.

The latest internationally recognised guidelines published by Scientific Committee on Emerging and Newly Identified Health Risks (SCENIHR) concluded that, based on all the current evidence, the threshold blood metal ion concentration for clinical concern is expected to be within the range of 2–7 μg/L, but exact levels have yet to be determined [5]. As such, it is imperative to realise that metal ion analysis should not be used in isolation when screening or investigating patients with MoM hip implants. Clinicians must carefully consider clinical examination findings, blood test results and radiologic investigations when determining the likelihood of a failing prosthesis. We have interpreted the SCENIHR final opinion to suggest that patients with blood metal ion concentrations >7 μg/L or those between 2 and 7 μg/L where there are other concerns, including clinical or radiologic abnormalities, should be monitored more closely with repeat testing and regular follow-up. Patients who fall into this group who are symptomatic should also be considered for revision surgery.

As metal ions are mainly excreted via the kidneys, individual patient factors can cause urine metal ion concentrations to vary greatly. As such, the unreliability of urine metal ion testing means that it should not be used for patient investigation or follow-up. In addition, because metal ion concentrations differ between serum and whole blood, it is important that serial tests are performed on similar samples and within the same laboratory to minimise variability in results. There should be standardised protocols in place for collection of serum or blood samples as needle type and collection techniques can change ion concentrations.

Systemic metal ion levels have been shown to accurately correlate with particle release at a local level into synovial joint fluid [33]. Therefore blood metal ion levels may be used as an appropriate substitute for synovial fluid analysis. Periprosthetic deposition of metal ions can be seen on histological analysis of retrieved samples collected at the time of revision surgery. Because of the variability of ARMD, however, it is not currently recommended to make decisions on joint revision surgery based on histological grading of adverse reactions [5]. Additionally, measuring exact levels in periprosthetic tissues requires the use of spectrometry and is extremely challenging, as exposure of specimens to room air can alter readings due to high chromium concentrations in airborne dust [34].

## 10. How Useful Is Imaging in Investigating Adverse Soft Tissue Reactions?

### 10.1. Plain Radiography (X-ray)

Plain radiography should be completed in all symptomatic patients with MoM bearing hip replacements and resurfacings in order to rule out periprosthetic fractures and acute dislocation. Beyond that, however, plain radiography is of little use as it tends to underestimate the prevalence of pseudotumours and can therefore falsely reassure clinicians [18].

### 10.2. Ultrasound Scanning (USS)

Ultrasound is a low cost, safe and readily available imaging modality that has better visualisation of joint effusions and tendinous pathologies compared with MRI [35]. Because it is less sensitive for detection of pseudotumours and muscle atrophy, however, it has been suggested that ultrasound only be used when metal artefact reduction sequence MRI is poorly tolerated, contraindicated or unavailable [35]. Furthermore, as ultrasound is operator dependent, an experienced musculoskeletal radiologist is required to accurately interpret the images.

### 10.3. Computed Tomography (CT)

While CT gives better bony resolution than plain radiography, because osteolysis is a late presentation in ARMD, CT is not suitable as an imaging modality for screening patients [36].

### 10.4. Magnetic Resonance Imaging (MRI)

With the addition of metal artefact reduction sequence (MARS), MRI has quickly become one of the most popular imaging modalities for visualising pseudotumours and muscle atrophy. When used as a screening tool, it can also detect soft tissue inflammatory reactions in asymptomatic patients [37]. Due to its high specificity and sensitivity for detection of these reactions and its versatility with assisting pre-operative planning and longitudinal comparison, MARS MRI has been recommended over ultrasound as the first-line modality for assessment of periprosthetic soft tissues in patients with MoM implants [35].

## 11. What about Pathological Examination of Retrieved Tissue?

At the time of revision surgery, periprosthetic tissue samples should be sent off for microbiological and histological examination in order to confirm the cause of implant failure. Frank pus seen at the time of revision, along with histological evidence of elevated neutrophils and microbiological culture of a pathologic organism suggests an infective cause. When this is ruled out, histological examination can focus on identifying patterns of aseptic failure secondary to ARMD. This tissue retrieval and analysis is essential as it helps characterise the pathogenesis of this complex reaction. Aseptic failure of MoM bearings was first described in terms of perivascular infiltration of lymphocytes, the presence of plasma cells and macrophages containing metal particles and severe ulceration of tissue surfaces [38]. 

Further research has determined that the spectrum of histological change seen in ARMD is thought to relate to the amount of wear of the implant. When implants are revised for suspected high wear, tissue samples are more likely to exhibit fewer lymphocytes, but more macrophages and metal particles [39]. When there is no evidence of high component wear, metal hypersensitivity should be considered. Metal hypersensitivity in MoM hip arthroplasty presents in a histologically similar manner, however, studies report increased lymphocytes, the addition of eosinophilic granulocytes and fewer macrophages and metal particles [39,40]. This is in addition to evidence of minimal component wear on the retrieved prosthesis.

## 12. When Is Revision Surgery Appropriate?

The SCENIHR has reinforced that there is no consensus on the appropriate timing of revision surgery for adverse reactions to metal debris in patients with MoM implants [5]. They have recommended that revision should be considered when symptoms become persistent, unmanageable and progressive. Furthermore, any patient exhibiting progressive osteolysis, large or expanding pseudotumours, progressive neck thinning or excessively high metal ion concentrations should also be considered for revision surgery.

The natural history of pseudotumours in asymptomatic MoM hip replacements remains largely unclear. Almousa *et al*. found that the majority increase in size over time with the occasional remission of small masses [19]. On the other hand, a longitudinal study found no change in pseudotumour size in 14 hips over 8 months [41]. After revision surgery, however, pseudotumours generally disappear.

## 13. What Are the Outcomes of Revision Surgery?

The outcomes of revision surgery are closely related to the indications for revision. In MoM hip resurfacing, the outcomes of revision for inflammatory pseudotumour are generally poor, with up to 50% of patients encountering major complications and a third requiring further revision [42]. Revision for reasons other than inflammatory pseudotumour, however, carries a much better outcome, similar to that after primary total hip replacement.

A small case series has reported complication rates of 68% after revision of Durom MoM THA for ARMD. The most common complications reported were dislocation, recurrence of ARMD and aseptic cup loosening [43]. The outcome of revision surgery of MoM bearing implants for ARMD seems to be related to the amount of tissue destruction, and hence the degree of subsequent surgical debridement required at the time of revision. A greater degree of debridement would lead to a less stable joint, increasing the risk of dislocation. As such, it is important to closely monitor patients with any radiological abnormality in order to offer revision surgery before extensive tissue destruction occurs.

Although the rate of recurrence of ARMD has not been established, a recent systematic review reported that ARMD recurrence was the second most common complication requiring re-revision, after dislocation [44]. When revising MoM implants, it is recommended to utilise a non-MoM articulation such as ceramic-on-ceramic or ceramic-on-polyethylene to reduce local metal ion release and hence the chance of recurrence of ARMD. Poorer clinical and/or functional outcomes have been documented in patients revised using MoM articulations [44]. Furthermore, recurrences of ARMD have been seen in patients revised using MoP bearings [43]. As this is thought to be related to wear debris released at the trunnion, the use of a ceramic head for revision is recommended to eliminate this risk.

Surgeons must ensure the entire ARMD lesion is excised and the metal debris completely removed. Failure to do so may contribute to persistence or recurrence of ARMD post revision. As such, resection of ARMD and pseudotumours has been likened to an oncological procedure where the surgeon should aim for clear resection margins to reduce the risk of recurrence [44]. This is often difficult, however, as pseudotumours can pass through tissue planes, extend into the pelvis and even involve vital neurovascular structures. In these cases, assistance should be sought from experienced vascular surgeons and/or pelvic reconstruction specialists.

## 14. Conclusion

The vast majority of patients with metal-on-metal hip replacements and resurfacings are unlikely to encounter any complications in the short to medium term as a result of their implants. Even with this knowledge, however, clinicians must remain vigilant and be thorough with their assessment and investigation of patients with these types of implants, regardless of whether or not a patient is symptomatic. Early diagnosis of adverse reactions and appropriate treatment for failing prostheses is essential for limiting the extent of soft tissue destruction and osteolysis that can occur as a result of adverse reactions to these implants.
